# Non-immersive virtual reality telerehabilitation for motor accuracy and precision in individuals with Cerebral Palsy: A non-randomized clinical trial

**DOI:** 10.1371/journal.pone.0343934

**Published:** 2026-03-13

**Authors:** Paula Lumy da Silva, Elisa de Jesus Valenzuela, Mariana Giovanelli de Carvalho, Anne Michelli Gomes Gonçalves Fontes, Juliana Perez Weingartner, Talita Dias da Silva-Magalhães, Íbis Ariana Peña de Moraes, Helen Dawes, Eduardo Dati Dias, Carlos Bandeira de Mello Monteiro

**Affiliations:** 1 Department of Physiotherapy, Fundação Hermínio Ometto (FHO), São Paulo, Brazil; 2 Postgraduate Program in Rehabilitation Sciences, School of Medicine, University of São Paulo (USP), São Paulo, Brazil; 3 Research Group and Technological Applications in Rehabilitation of the School of Arts, Sciences and Humanities of the University of São Paulo (PATER EACH-USP), São Paulo, Brazil; 4 Faculty of Health and Life Sciences, University of Exeter, Exeter, United Kingdom; 5 Postgraduate Program in Rehabilitation Sciences and Physical and Functional Performance, Federal University of Juiz de Fora Campus Governador Valadares (UFJF-GV), Minas Gerais, Brazil; 6 National Institute for Health and Care Research, Exeter Biomedical Research Centre and HealthTech Research Centre, College of Medicine and Health, University of Exeter, Exeter, United Kingdom; University rehabilitation institute, SLOVENIA

## Abstract

This study aimed to describe motor performance, accuracy and precision, in individuals with Cerebral Palsy (CP) compared to typically developing individuals (controls), and to examine longitudinal changes during a non-immersive virtual reality (VR) telerehabilitation protocol. Methods: The final sample included, 38 with CP and 21 controls. Controls completed a single practice session, while 29 participants with CP completed the 10-day home-based protocol, and 20 returned for a follow-up session to assess retention. Analyses followed the intention-to-treat principle, including all 38 CP participants. The intervention used the MoveHero game, performed two to three times per week, with sessions lasting at least 9 minutes under remote therapist supervision. Motor performance was assessed in a coincident timing task using absolute error (AE, accuracy), variable error (VE, precision), and percentage of hits. The study was registered in the Brazilian Clinical Trials Registry (ReBec: RBR-4kf52bv). Results: The CP group had higher mean AE and VE values than controls but showed progressive improvements across practice days. Mean accuracy improved across most target positions (p < 0.05), with mean AE decreasing from 1350 ms on Day 1–895 ms on the best practice day. Controls had a mean AE of 584 ms, and participants with CP achieved similar performance in two of the four target positions. Mean VE improved notably for Target 3 (1124 ms on Day 1–811 ms on Day 9, p = 0.007), and the mean percentage of correct responses increased from 45.8% to 58.5% (p = 0.019), while controls reached 69.1%. No significant differences were found among GMFCS subgroups. Gains were maintained at follow-up (p > 0.05). Conclusion: Home-based non-immersive VR telerehabilitation using MoveHero effectively enhanced upper-limb motor performance in individuals with CP, regardless of functional level. These findings support VR as an accessible, motivating, and adaptable tool for promoting motor learning and continuity of care in home-based rehabilitation.

## Introduction

Cerebral Palsy (CP) refers to a group of permanent disturbances of movement and postural development, causing activity limitations attributed to non-progressive disturbances which occurred during fetal or infant brain development. In addition to motor disorders, CP is often accompanied by disturbances in sensation, perception, cognition, communication, behavior, epilepsy, and secondary musculoskeletal problems [1].

Due to the presence of these dysfunctions, it is essential that individuals with CP have continuous access to rehabilitation services, in order to maintain and improve motor skills, activities of daily living, and prevent further complications, such as orthopedic deformities and physical deconditioning to ensure a better quality of life [2].

A systematic review presented different intervention possibilities for individuals with cerebral palsy [3]. In addition, evidence on the use of virtual reality (VR) has been growing in clinical practice. VR has been shown to improve hand manipulation, increase manual reach speed, dexterity [4], manual performance, motor skills, and activities of daily living [5], reduce gait onset time, and increase mobility [6].

An important use of new technological tools, such as VR, is to offer interesting opportunities to maintain the treatment of individuals with CP through home-based telerehabilitation (HBTR) [7]. HBTR offers the possibility of streamlining rehabilitation services, by reducing therapist time [8], and providing the opportunity to increase the frequency of skills training for people who have difficulty regularly attending rehabilitation centers [9].

Thus, Silva et al. [10] designed a cross-sectional study to evaluate the feasibility and potential benefits of an HBTR program incorporating VR and serious gaming for individuals with cerebral palsy (CP) and a typically developing (TD) group. Using a coincident timing game, the study assessed improvements in task performance for both groups. The findings revealed that both CP and TD demonstrated enhanced performance during game practice, highlighting the promising potential of HBTR as an effective rehabilitation approach.

Considering the above, the present study (based on da Silva et al. [10]) aimed to observe performance in a longitudinal protocol using a serious gaming platform implemented and evaluated remotely in individuals with CP during HBTR, while also considering the different functional levels of CP. Thus, a 10-day protocol with a coincident timing game was adopted: (1) to estimate the extent and timing of the improvements in task performance in response to a long-term telerehabilitation program in individuals with CP; (2) to estimate the extent of differences between Cerebral Palsy and Typical Development (control group); and (3) to describe the response in task performance among individuals with varying levels of gross motor function within the individuals with CP.

We hypothesized that individuals with CP would demonstrate improved task performance through practice, with the best performance observed on the final day. Additionally, we expected individuals with CP to perform worse compared to the control group, with greater difficulties observed in individuals with more severe motor impairments. If confirmed, these findings would highlight the relevance of using HBTR for individuals with CP.

## Materials and methods

### Study design

This study was approved by the Research Ethics Committee of the Hospital das Clínicas, School of Medicine, University of São Paulo (protocol: 38563420.0.0000.0068), and registered in the Brazilian Clinical Trials Registry (ReBEC) under the number RBR-4kf52bv. This is a non-randomized controlled clinical trial conducted between January 20, 2021, and March 30, 2024. The study included individuals with cerebral palsy (CP) and typically developing (TD) individuals, who composed the control group. Participants were recruited through video calls, multiple neurorehabilitation clinics in São Paulo, social networks, and therapist referrals. The study was carried out entirely in a remote format, with researchers interacting with participants via telephone calls, messaging applications, and videoconferencing. All parents or legal guardians provided written informed consent by signing the Free and Informed Consent Form, and minors under 18 years of age signed the Assent Form. Data collection began only after all required forms were duly signed. This study follows the guidelines and recommendations of the Consolidated Standards of Reporting Trials (CONSORT) [11,12], which are detailed in the Supporting Information (S1 Checklist and S2 File).

### Inclusion and exclusion criteria

For both the CP and control groups, participation required: (1) consent through the signing of the assent term by the participant and the Free and Informed Consent Form by the parents or legal guardians (for minors); (2) access to a technological device (e.g., smartphone) for communication with the research team; and (3) the ability to understand and perform the MoveHero task after demonstration and brief familiarization (based on the criteria established by Almeida et al. [13]).

Participants in the CP group were additionally required to have a clinical diagnosis of cerebral palsy, of either sex, and not present motor impairments that prevented performing the virtual task, even after adjusting distance and camera positioning, such as severe spasticity, contractures, or limited range of motion that hindered interaction with the MoveHero system. Control group participants were additionally required to have no motor or neurological disorders, to be within a comparable age range to the CP group, and to be predominantly male to approximate the sex distribution of the CP group. Participants were excluded if they were unable to complete the protocol due to technological issues (e.g., internet instability or device malfunction).

### Participants

**Fig 1:**
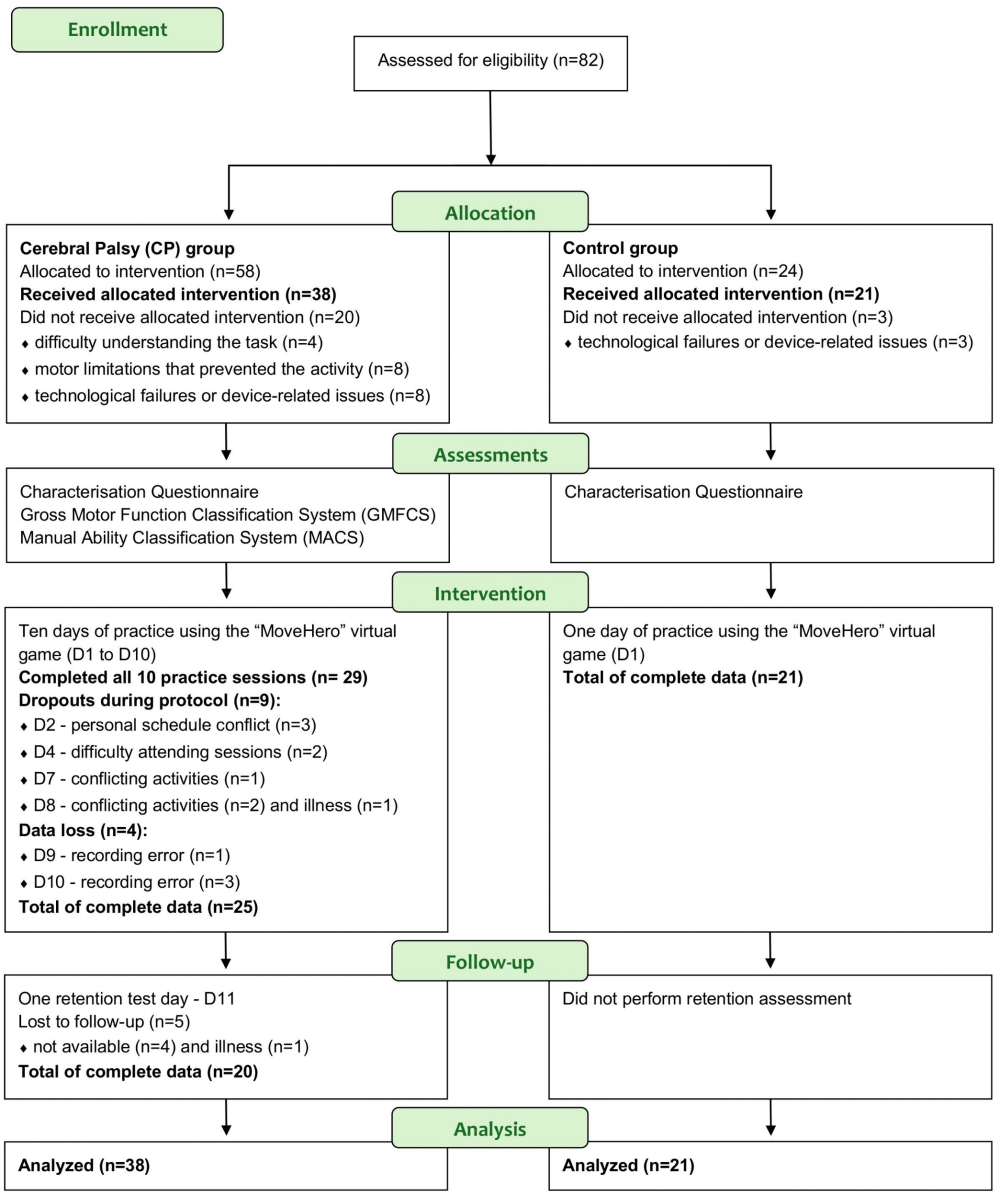
CONSORT flow diagram.

A total of 82 participants were initially included (Fig 1). After 24 exclusions, the final sample consisted of 59 participants (38 with CP and 21 with TD). The numbers for each group are presented below: 58 participants with CP were initially assessed for eligibility, and included, recruited through video calls, multiple neurorehabilitation clinics in São Paulo, social networks, and therapist referrals. Twenty participants with CP were excluded based on: four due to difficulty understanding the task, eight due to motor limitations that prevented the use of MoveHero, and eight due to technological failures or device-related issues. The final CP group therefore comprised 38 participants who met all study criteria. A total of ten practice sessions were conducted using the MoveHero virtual game (Days 1–10). Twenty-nine participants completed all ten sessions of the intervention.

In addition, 24 typically developed (TD) individuals were recruited through convenience sampling to form the control group, of which 3 were excluded due to technological failures or device-related issues. Resulting in a total sample of 59 participants (38 CP and 21 TD). Although formal matching was not performed, age was considered as a guiding factor in the selection of control participants to ensure comparability between groups, and efforts were made to achieve a sex distribution comparable to that of the CP group.

### Procedures

After inclusion, participants from both groups completed the Characterization Questionnaire, which contained information on age, gender, weight, and height. For the CP group, the Gross Motor Function Classification System (GMFCS) and the Manual Ability Classification System (MACS) were applied to assess motor difficulties. The GMFCS classifies the severity of motor impairments based on self-initiated movement, focusing on sitting, transfers, and mobility. It comprises five levels (I–V), ranging from Level I, indicating independent mobility without limitations, to Level V, representing severe motor restrictions even with assistive technology [14]. The MACS assesses how participants use their hands to handle objects in daily activities. It also includes five levels (I–V), ranging from Level I, which indicates effective and independent handling of objects, to Level V, representing an inability to handle objects and severely limited manual abilities [15].

Following the assessments, participants received detailed instructions regarding the game settings, level selection, degree of difficulty, camera activation, and visual effects. The participant, accompanied by a caregiver, was instructed to place the notebook running the game on a table approximately 1.5 meters away and to position a smartphone beside it to receive real-time guidance via video call. The researchers explained the task both verbally and through gestures, and the caregiver was asked to operate the game according to these instructions. After the demonstration, the caregiver assisted the participant in preparation and adjusted the smartphone position so the researcher could monitor performance during practice.

The participant’s posture during the task was determined according to their GMFCS level: individuals at levels I and II were encouraged to perform the task while standing; those at level III could perform it either standing with support or seated, depending on balance and safety assessments; and participants at levels IV and V performed the task in their wheelchair.

**Fig 2:**
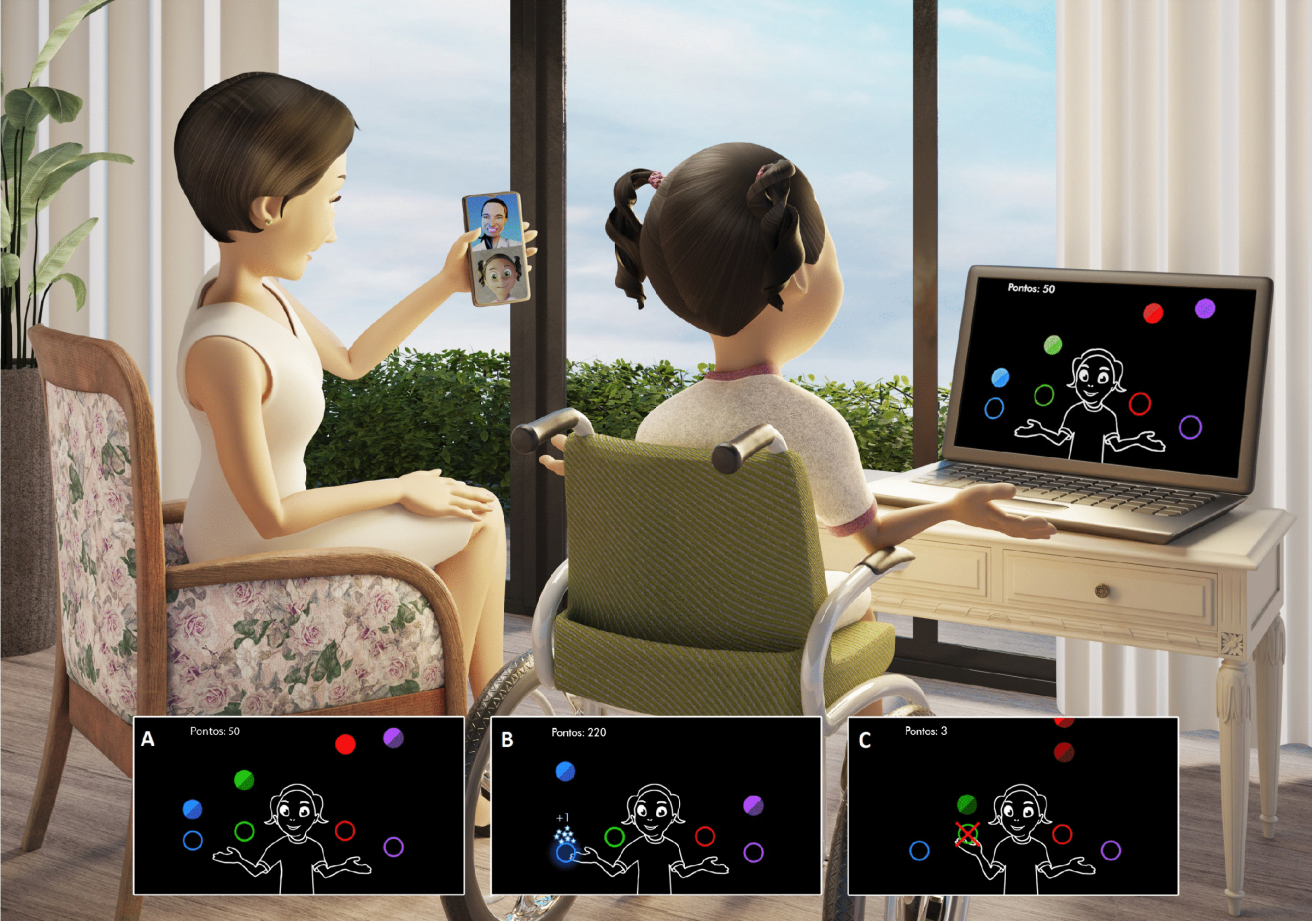
Participant positioning and game design. The participant was seated approximately 1.5 meters away from the computer screen and moved their arms to capture the spheres. The caregiver assisted the therapist through a video call, allowing instructions to be given in real time. (A) Task targets illustrated using colored circles: the blue circle represents position 1, the green circle represents position 2, the red circle represents position 3, and the purple circle represents position 4. (B) Illustration showing a successful hit by the participant, with the targeted sphere highlighted. (C) Illustration depicting a missed attempt by the participant, indicated by a red X on the sphere. This figure was created specifically for this publication. Republished from: Bach P. 2025. Graphic Design Work. Republished under a CC BY license, with permission from Paulo Bach, original copyright 2025.

The participant was instructed to wait for the first sphere to appear on the screen, while the caregiver initiated the game. Once the first sphere appeared, the participant began moving their hand toward the target (Fig 2).

### Motor performance intervention – MoveHero

MoveHero is a coincident timing task game developed at the School of Arts, Sciences, and Humanities of the University of São Paulo [16–19]. The game, freely available in Portuguese and English (https://movehero.com.br/en/), was used to assess and train motor coordination (Fig 2). A caregiver created an account for each participant, ensuring data security and investigator access. MoveHero displays falling spheres across four imaginary lines on the screen, synchronized with a musical rhythm selected by the researcher. Participants use upper limb movements to intercept spheres at precise moments, following a coincident timing task format. A webcam captures movements, converting them into actions by an on-screen avatar, allowing interaction without physical contact.

The game features four target positions represented by colored circles: Position 1 (blue, lateral left), Position 2 (green, central left), Position 3 (red, central right), and Position 4 (purple, lateral right). Participants, moved their upper limbs and trunk to reach the targets as the spheres fell. The platform provides real-time feedback, successful hits turn the target blue with stars, while misses are indicated by a red ‘X’. A numerical score (+1) appears beside each correct response, and the total score is displayed in the upper-left corner. The game’s adjustable difficulty levels ensure accessibility for individuals with varying motor abilities. For further details on MoveHero’s application, see Silva et al. [10].

### Intervention protocol

The intervention protocol consisted of 10 days of training practice (D1 to D10), conducted two to three times per week, with each session lasting at least 9 minutes, and included continuous remote contact between the therapists and patients. To ensure adherence to the telerehabilitation protocol, therapists provided real-time supervision and information during all training sessions. Only the CP group completed all 10 days of practice, while the control group performed a single practice session to provide comparative data. Each practice day comprised three 3-minute matches, referred to as Match 1 (M1), Match 2 (M2), and Match 3 (M3). Motor performance was evaluated daily using the absolute error (AE), variable error (VE) and percentage of hits, parameters automatically generated by the MoveHero platform. After completing the 10 days of practice (D10), participants in the CP group underwent a 15- to 30-day washout period, followed by a single follow-up session to assess retention (D11).

### Statistical analysis

The characterization of the individuals is presented as percentages for qualitative variables and means (M) and standard deviations (SD) for quantitative variables. Regarding the dependent variables in the “MoveHero” task, error measures were considered, defined as the difference between the moment the sphere reached the target (time of arrival) and the time when the participant’s gesture was registered, in milliseconds (see details in Silva et al. [10]). From these primary error measurements, AE and VE were derived. AE reflects movement accuracy, representing the absolute difference between the expected and actual response time, regardless of whether the response was early or late. VE reflects movement precision, indicating the consistency of a participant’s responses across multiple trials, independent of accuracy. The percentage of successful hits (spheres intercepted) during the game was also considered as a dependent variable [20].

Data were analyzed according to the intention-to-treat principle. The time-dependent error variables (AE and VE) were analyzed using Linear Mixed Models (LMM) test with a 4 (Target position: P1 - P4) by 3 (Matches: M1 - M3) by 11 (Days of practice: D1 - D11) design, with repeated measures in all factors. The number of hits was analyzed using LMM with a 3 (Matches: M1 -M3) by 11 (Practice days: D1 to D11) design, with repeated measures in all factors. Practice retention was assessed by comparing performance on D10 with D11.

Additionally, we compared the performance of the Control group, which completed only a single practice session, with both the first day (D1) of the CP group and CP group’s best performance day. The best day was defined separately for each outcome: for AE and VE, it corresponded to the day with the lowest average errors, and for the percentage of successful hits, it corresponded to the day with the highest average percentage of hits.

Data presented in the graphs are expressed as means ± standard error. Statistical analyses were performed using the Statistical Package for the Social Sciences (SPSS), version 26.0. A p-value of <0.05 was considered statistically significant, while p-values between 0.05 and 0.08 were considered indicative of a marginal difference. All data from this study are publicly available [21].

## Results

The study initially recruited 82 participants, including 58 individuals in the CP group and 24 in the control group. Following the application of exclusion criteria, 24 participants were excluded, resulting in a final sample of 59 participants: 38 in the CP group and 21 in the control group. All control participants performed a single practice session of the experimental task. Among the CP group, all 38 participants began the training protocol and completed most of the practice sessions, with 29 completing the full 10-day protocol. Data from four participants were lost due to recording errors on Days 9 and 10, leaving 25 participants with complete data for analysis, and 20 of them returned for the retention test on Day 11 (Fig 1). Data from all 38 CP participants were included in the overall analyses according to the intention-to-treat principle, regardless of protocol completion (Table 1).

**Table 1:** Demographic data.

VARIABLES			p-value
	CP	Control	
	*n = 38*	*n = 21*	
	*Mean±SD*	*Mean±SD*	
Age	17.6 ± 11.1	18.5 ± 9.4	0.759
Weight	46.7 ± 16.7	59.5 ± 23.8	0.036
Height	1.47 ± 0.18	1.59 ± 0.21	0.033
BMI	21.3 ± 5.2	22.6 ± 5.5	0.383
	*n (%)*	*n (%)*	
**SEX**			
Male	24 (63.2)	13 (61.9)	0.571
Female	14 (36.8)	8 (38.1)	
**MACS**			
*I*	18 (47.4)	–	
*II*	11 (28.9)	–	
*III*	6 (15.8)	–	
*IV*	3 (7.9)	–	
*V*	0 (0.0)	–	
**GMFCS**			
*I*	8 (21.1)	–	
*II*	8 (21.1)	–	
*III*	12 (31.6)	–	
*IV*	7 (18.4)	–	
*V*	3 (7.9)	–	

CP: Cerebral Palsy; SD: Standard deviation; BMI: Body mass index; MACS: Manual Ability Classification System; GMFCS: Gross Motor Function Classification System.

### Motor performance with MoveHero software

The motor performance of the upper limbs was measured from the AE and VE measured through a *coincident timing task* using a VR game. The error was defined as the time difference between the time when the target sphere should have been hit (arrival time) and the time when the participant’s hand reached the target and was registered. The AE expresses accuracy, the VE indicates variability in performance, and the reduction in errors indicates improvement in performance. All the values are presented as mean values, and additional data, such as standard error, confidence intervals and effect sizes are presented in the Supporting Information (S3 Table).

#### Absolute error (AE).

We decided to present the AE considering a comparison between all 4 target positions (P1 - P4) for the CP group during the days of practice. Moreover, we organized a comparison between groups using the first day of practice (i.e., CP and controls first day of practice) and CP best day of practice (i.e., CP best day of practice compared with Control group on the first day – to identify if CP can present similar performance to Control group on the best performance day).

Position 1: a marginal effect was found for the Days factor (p = 0.056). Post-hoc comparisons showed that on the first day of practice (D1) the participants had significantly higher AE (1278 ms) when compared to the other days (D2 = 923 ms, p = 0.007; D3 = 838 ms, p = 0.001; D4 = 969 ms, p = 0.022; D5 = 977 ms, p = 0.025; D6 = 922 ms, p = 0.008; D7 = 809 ms, p = 0.001; D8 = 847 ms, p = 0.002; D9 = 964 ms, p = 0.025; D11 = 902, p = 0.016) with a marginal difference to day 10 (1023 ms, p = 0.078). Therefore, considering the shortest execution time, D7 was the best day of practice. In the comparison with the Control group, a significant difference was found on D1 (488 ms; p < 0.001) with higher AE in the CP group, and when compared with the best performance day (D7), we found a marginal difference (p = 0.068), with higher AE in the CP group. Participants showed task retention, observed by comparing D10 with D11 (p = 0.472).

Position 2: A main effect was found for the Days factor (p = 0.034). Post-hoc comparisons showed that on the first day of practice (D1) the participants presented significantly higher AE (1402 ms) when compared to two other days (D8 = 970 ms, p = 0.005 and D9 = 1017 ms, p = 0.013). Therefore, considering the shortest execution time, D8 was the best day of practice. In the comparison with the Control group, a significant difference was found on D1 (776 ms; p = 0.005) with higher AE in the CP group, and when compared with the day of best day of practice D8, we did not find a statistical difference (p = 0.305), which demonstrates that the CP group reached values equivalent to the Control group in this position. Participants showed task retention (D10 versus D11, p = 0.734).

Position 3: A main effect was found for the Days factor (p = 0.004). Post-hoc comparisons showed a decrease in AE over the days of practice, on D1 (1691 ms) when compared to all other days (D2 = 1226 ms, p = 0.002; D3 = 1227 ms, p = 0.003; D4 = 1253 ms, p = 0.005; D5 = 1205 ms, p = 0.002; D6 = 1090 ms, p < 0.001; D7 = 1154 ms, p = 0.001; D8 = 1160 ms, p = 0.001; D9 = 1282 ms, p = 0.011; D10 = 1099, p < 0.001; D11 = 954, p < 0.001). Therefore, considering the shortest execution time, D6 was the best day of practice. In the comparison with the control group, a significant difference was found on D1 (716 ms; p = 0.003), when compared with the day of best performance, D6, with a marginal difference (p = 0.070), behaving similarly to the performance in position 1. Participants showed task retention (D10 versus D11, p = 0.465).

Position 4: A main effect was found for the Days factor (p = 0.005) and for the Matches factor (p = 0.006). Post-hoc comparisons show that in the Matches, the participants decreased AE from the first M1 (1030 ms) to the other matches (M2 = 858 ms, p = 0.014; M3 = 818 ms, p = 002). In addition, there was a decrease in AE over the days of practice, with D1 having the longest error time (1254 ms) when compared to all other days (D2 = 881 ms, p = 0.002; D3 = 935 ms, p = 0.010; D4 = 880 ms, p = 0.003; D5 = 997 ms, p = 0.036; D6 = 937 ms, p = 0.010; D7 = 847 ms, p = 0.001; D8 = 810 ms, p < 0.001; D9 = 711 ms, p < 0.001; D10 = 786, p < 0.001; D11 = 886, p = 0.009). On D1 there was a decrease in AE between the first and last matches (M1 = 1455 ms, M3 = 1018 ms; p = 0.034), while on D2 there was a marginal decrease between the first (M1 = 1146 ms) and the other matches (M2 = 742 ms, p = 0.052; M3 = 756 ms, p = 0.070). Therefore, considering the shortest execution time, D9 was the best day of practice. In the comparison with the Control group, a significant difference was found on D1 (359 ms; p = 0.001), when compared with the day of best performance, D9, with a significant difference (p = 0.003), in which the improvement in this position did not reach the values of the Control group. Participants showed task retention (D10 versus D11, p = 0.515).

**Fig 3:**
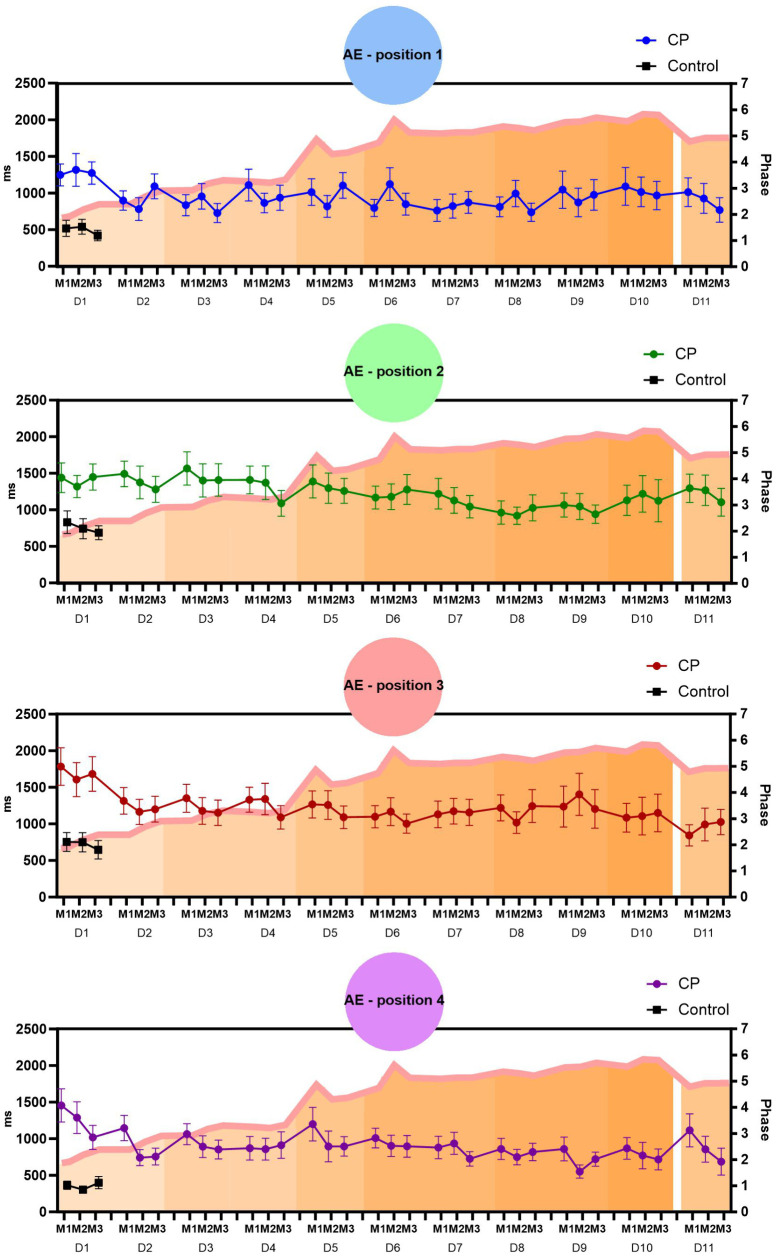
Representation of mean and standard error of the AE in the 3 matches (M1-M3) and in the four positions (P1 - P4) using MoveHero software over the 11 days of practice (D1 – D11).

Fig 3 presents the mean and standard error of the AE in the 3 matches (M1-M3) and in the four positions (P1 - P4) using MoveHero software over the 11 days of practice (D1 – D11).

#### Variable error (VE).

Positions 1 and 2: No main effects were found, but post-hoc comparisons identified a decrease in the VE at position 2 (central left side) on D2 between M1 and M2 (M1 = 1286 ms, M2 = 943 ms, p = 0.024). When comparing performance with the Control group, in position 1, the CP group presented a higher VE on D1 (867 ms) than the Control group (565 ms). At position 2, the CP group presented a marginal difference (1120 ms) compared to the Control group (828 ms; p = 0.059). Since there was no improvement in performance, no comparisons were performed with the CP group’s best day. Participants showed task retention in both positions (D10 versus D11, P1 p = 0.891 and P2 p = 0.752).

Position 3: A main effect was found for the Days factor (p = 0.007). Post-hoc comparisons showed a decrease in VE over the days of practice, from D1 (1124 ms) to D9 (811 ms, p = 0.001) and D11 (871 ms, p = 0.023). Therefore, considering the shortest execution time, D9 was the best day of practice. In the comparison with the Control group, a marginal difference was found on D1 (796 ms; p = 0.062), when compared with the day of best performance, D9, with no statistical difference (p = 0.932). Participants showed task retention (D10 versus D11, p = 0.422).

Position 4: No main effects were found. In comparison with the control group, a significant difference was found on D1 (PC = 763 ms; Control = 433 ms; p = 0.004), since there was no improvement in performance, no comparisons were performed with the PC group’s best day. Participants showed task retention (D10 versus D11, p = 0.066).

**Fig 4:**
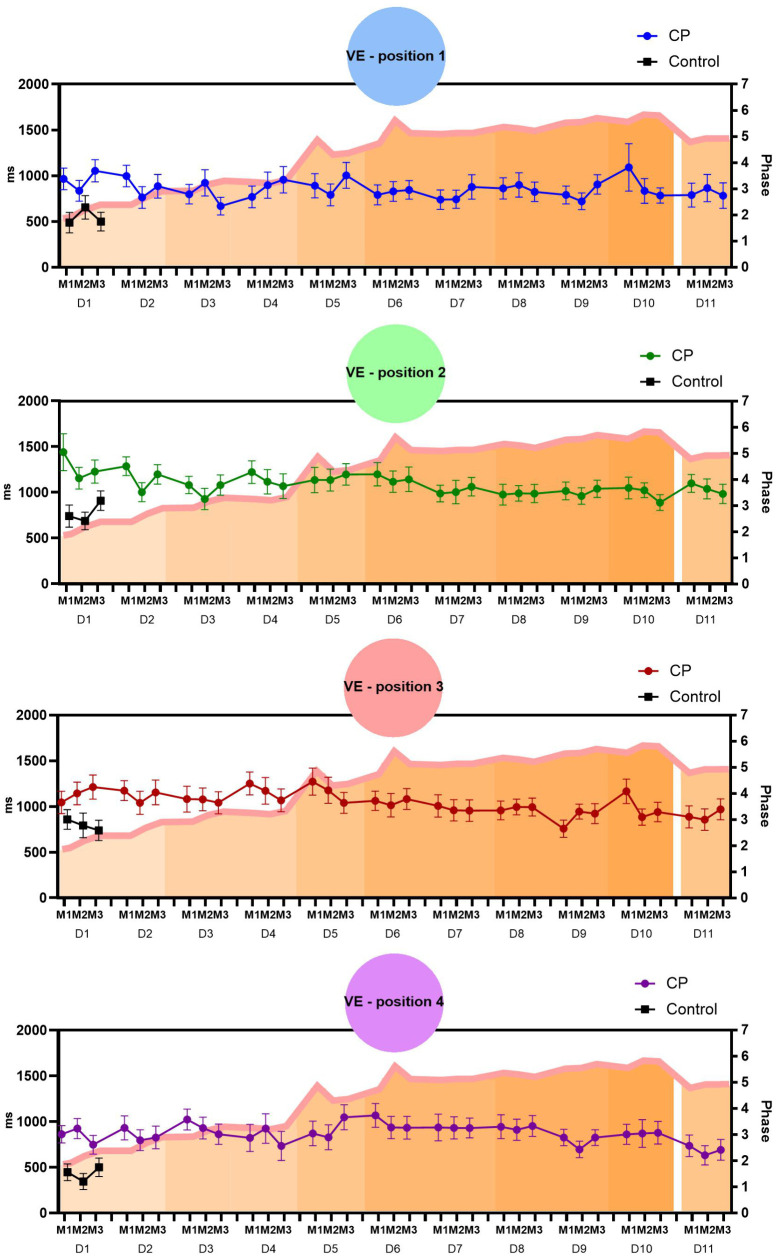
Representation of mean and standard error of the VE in the 3 matches (M1-M3) and in the four positions (P1 - P4) in the MoveHero software over the 11 days of practice (D1 – D11).

Fig 4 presents the mean and standard error of the VE in the 3 matches (M1-M3) and in the four positions (P1 - P4) in the MoveHero software over the 11 days of practice (D1 – D11).

#### Hits.

For the percentage of correct answers (% Correct answers), a main effect was found for the Days factor (p = 0.019). Post-hoc comparisons identified that on the first day, D1, the participants had the lowest percentage of hits (45.8%) when compared to the other days (D2 53.8%, p = 0.017; D3 52.8%, p = 0.041; D4 54.1%, p = 0.015; D5 56.4%, p = 0.002; D6 57.7%, p = 0.001; D7 57.0%, p = 0.001; D8 58.5%, p < 0.001; D9 55.0%, p = 0.010; D10 54.1%, p = 0.024), with the exception of D11, which showed no statistical difference (50.2%, p = 0.272).

**Fig 5:**
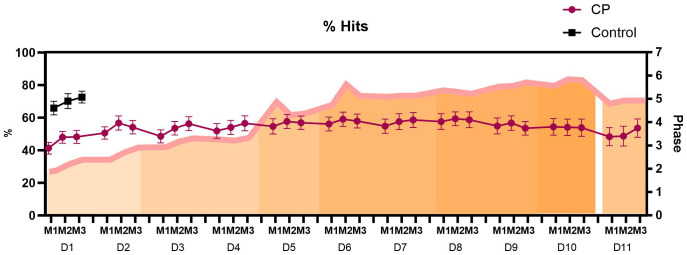
Representation of mean and standard error of the percentage of Hits in the 3 matches (M1-M3) in the MoveHero software over the 11 days of practice (D1 – D11).

Therefore, considering the shortest execution time, D8 was the best day of practice (Fig 5). In the comparison with the control group, a significant difference was found on D1 (69.1%; p < 0.001), when compared with the day of best performance D8, a statistical difference was maintained (p = 0.012). Participants showed task retention (D10 versus D11, p = 0.360).

### Comparison of motor performance according to GMFCS

**Fig 6:**
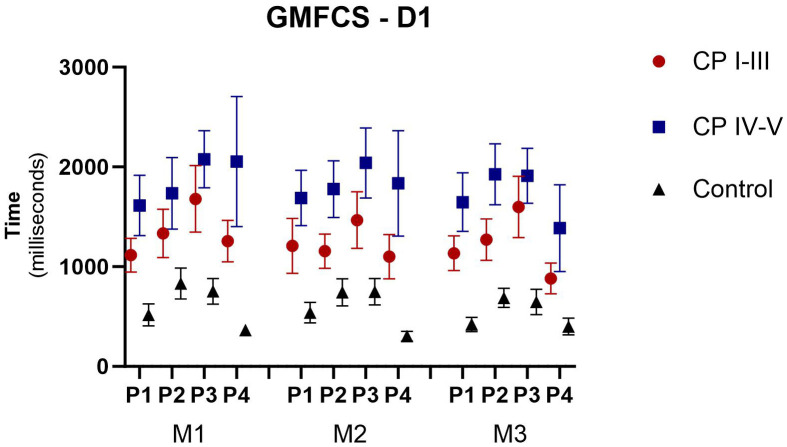
Representation of mean and standard error of the AE in the 3 matches (M1-M3) and 4 positions, subdivided by the GMFCS on D1.

To determine whether the GMFCS score influenced performance in the task, we subdivided the participants with CP into two groups, with levels I-III and levels IV-V, and compared them with the controls (Fig 6). The main effect was found between the groups (p = 0.016), in the analysis of D1, whereby in all positions and matches, groups I-III and IV-V presented higher AE than the control group. No statistical differences were found between CP groups (I-III versus IV-V).

## Discussion

The main objective of the present study was to analyze the motor performance of individuals with CP who participated in a long-term protocol of home telerehabilitation using a virtual reality game. As we hypothesized, the CP group presented more difficulty in practicing the task than the controls. Comparing the first day with the last day of practice all participants with CP improved their performance in both AE and VE. However, and differently from our hypothesis, the day of best performance varied depending on the position of the task and there was no difference within the CP individuals with different motor function levels. These results will be discussed in more detail below:

### Comparison between participants in the CP group and control group

The typically developed individuals performed better than the CP group in most positions and matches (i.e., control group practiced the task only on the first day to collect normal baseline information). This finding is in agreement with de Mello Monteiro et al. [22] and Prado et al. [23] who compared motor learning between individuals with CP and their typically developing peers in a coincident timing task in non-immersive VR. As there is an increase in attempts at the task (repetition), both typical children and those with CP presented improved performance in the task, however, despite the improvement, children with CP did not achieve the same results as typical children in the acquisition. According to Cheng et al. [24], children with CP, when compared to typical children, present less variability in acquisition and higher retention when submitted to non-immersive VR. The variability observed was consistent with the challenges expected during the task and was related to motor impairment and age.

The difficulties of CP, such as permanent neurological impairment [25], associated with significant sensorimotor dysfunction [26], muscle weakness [27], high levels of coactivation [27], abnormal muscle recruitment with spasticity [28,29], and slowness of movement [10] impair the performance of individuals with CP. According to Burtner et al. [30] changes in muscle tone, perception, range of motion, and muscle strength, associated with the precision and speed requirements of the upper limbs, end up affecting the functional abilities of individuals with CP.

### Days of practice in the CP group

As hypothesized, there was improvement in the motor performance of individuals with CP throughout the protocol considering AE, VE, and correct hits. The results showed that the best improvement was achieved on different days in each target position and varied between the 6th and 9th day of practice, with a decrease in performance on the 10th day but with retention to the 11th day (i.e., we identified retention of performance in all target positions). Thus, we have three factors to discuss:

#### Improvement in performance.

One interesting result was that the improvement in performance was only identified after the 5th day of practice. This suggests that practicing the virtual reality task for more than 5 days influences the improvement in motor performance.

Some studies found benefits of practicing VR games. Choi et al. [31], demonstrated that VR enhances motor learning by facilitating implicit learning through enjoyable gaming experiences and promoting focused attention. The incorporation of gamification in VR leads to heightened motivation, as noted by Cano Porras et al. [32], and superior engagement and emotional involvement compared to traditional exercises, according to Bonnechère et al. [33]. Therefore, benefits were found in manual function [34,35], postural control [36], balance and motor skills [37], and activities of daily living [38].

Building on these findings, it is important to note that the improvements observed in the current study occurred in a coincident timing task, which is specifically designed to train the temporal coordination of movements. Participants with cerebral palsy demonstrated gains in this task across the training protocol, highlighting their ability to adapt and refine motor timing through repeated practice in a VR environment. Similar improvements have been observed in previous studies; as demonstrated by Johansson et al. [39], who reported long-term gains in upper-limb timing through synchronized training in children with CP, while Accogli et al. [40] emphasized the role of perceptual and motor timing in facilitating motor learning. These findings suggest that task-specific VR practice can effectively target temporal aspects of motor control.

#### Performance plateau on Day 10.

Contrary to our initial hypothesis, performance on Day 10 of the protocol did not show further improvement compared with previous days, indicating a plateau rather than a decline. It was expected that after nine days of practice, participants would continue to show gains. Although task repetition is an important factor in skill acquisition [41], it is important to note that higher doses do not always translate into greater improvements [42], and the number of practice days should be carefully considered.

Engagement and motivation, which characterized the VR task, likely supported attention and performance improvements from Days 6–9; however, repeating the same task over multiple sessions may have limited further gains. Similar effects have been observed in VR-based rehabilitation for children with CP, where initial novelty and engagement decline as repetition makes the activity resemble conventional therapy. This underscores the importance of balancing task variability and providing the optimal “just-right challenge” to sustain motivation and skill improvement over time [4,23,43]. Greater variability of tasks could promote performance improvements over a longer period during VR rehabilitation [23].

This interpretation is consistent with findings by Sandlund et al. [44], who reported that children’s interest in games decreased somewhat over time. Similarly, Farr et al. [45] conducted a 12-week home-based intervention using Nintendo Wii Fit™, with two groups (one supported by physiotherapists and one without support, allowing children more freedom to choose games). Despite positive outcomes, a decline in engagement was observed by the seventh week, highlighting the need to consider motivation and task variety in designing VR rehabilitation protocols.

#### Retention.

Our results showed no difference between the 10th and 11th (i.e., retention after 15–30 days) days of practice, which supports the task retention. Although studies are scarce that evaluate the retention of skills with VR training [46], as well as the dose, number of repetitions, and the form of difficulty progression [47], some studies have reported the potential of VR to promote retention of the new skills acquired in the upper limbs using reaching tasks [41,48], range of movement [49], and the mobile maze task [50]. Thus we agree with the Systematic Review and Meta-Analysis about the Short- to Long-Term Effects of Virtual Reality on Motor Skills in individuals with cerebral palsy [51], which reports that VR devices developed specifically for therapy had greater potential to make acquisitions persistent in the medium or long term.

### Task-specific effects on precision

Although no direct comparison between target positions was performed, improvements in VE were only observed for Target 3, located closer to the right side. This pattern likely reflects the influence of task-specific spatial and biomechanical demands rather than inconsistency in learning. Targets that required greater movement amplitude and control may have posed additional challenges for individuals with cerebral palsy, limiting precision gains. Such findings are consistent with previous research showing that biomechanical constraints and movement complexity can significantly influence motor performance and learning in children with CP [52,53]. These results emphasize the importance of considering the spatial layout and required movement amplitudes when designing VR-based rehabilitation tasks to maximize precision improvements.

Task-specific demands are particularly relevant in timing and coordination tasks, where movement trajectories and required amplitudes interact with motor control abilities. Studies have demonstrated that children with CP show differential improvements depending on target distance and task complexity, highlighting the role of individualized task difficulty in motor learning [23,54]. Incorporating adaptive or progressively challenging target arrangements in VR interventions may enhance precision gains across a broader range of motor tasks, ensuring that participants can engage effectively without being limited by biomechanical constraints. These considerations support the use of VR as a flexible tool for rehabilitation, where task design can be optimized to each participant’s motor abilities and learning needs.

### Comparison between levels of individuals with CP

In analyzing motor performance across different GMFCS levels on the first day of practice, an interesting result emerged. It was initially hypothesized that individuals with more severe motor impairments would exhibit significantly poorer performance. However, no significant differences were observed between participants with higher (I, II, and III) and lower (IV and V) GMFCS levels on Day 1.

These findings suggest that at the outset of VR-based telerehabilitation, individuals with CP, regardless of their motor function level, were able to engage in the task at a similar performance level. While this does not provide direct evidence of differential responses to training over time, it highlights the accessibility and inclusivity of VR tasks, as they allow individuals across a broad spectrum of motor abilities to participate meaningfully.

This equitable performance may be explained by two main factors: **(a)** Adaptive Difficulty: The game used adjusted the task difficultyin real-time based on each participant’s performance, ensuring that individuals across all GMFCS levels were appropriately challenged. This allowed meaningfull engagement without excessive frustration or boredom. VR environments can be tailored to individual capabilities, allowing users with different levels of motor function to engage with tasks in an accessible and progressively challenging manner [51]. This personalization can facilitate optimal engagement and motivation at different functional levels, potentially leveling performance outcomes. It should also be taken into account that in addition to the severity of CP, the individual’s age and responsiveness, as well as the different technologies and designs used in VR can modify the effectiveness of VR interventions [38]. Regarding game designs, so-called commercial games are more limited [55], since depending on the degree of disability, individuals with CP may not be able to achieve a minimum score to progress in a given game [46]. In this sense, the VR game used in the current research, can be considered more suitable for different levels of CP, as it allows variation in the degree of difficulty in an individualized way. **(b)** Engagement and Motivation: interactive and individual levels of difficulty contribute to higher levels of engagement and motivation among participants, regardless of their level of motor function [56]. This increased motivation can facilitate repetition and practice for individuals with different levels of difficulty, which are crucial for motor learning and rehabilitation outcomes, developing autonomy, and providing improved choices and expectations that improve motivation and interest in the task [24,57].

While novelty and motivation effects were not formally measured in the current study, it is likely that the VR environment contributed to these factors. The interactive and gamified nature of the task may have enhanced participants’ initial interest and engagement, promoting sustained attention and effort during practice sessions.

### Limitations

While this study highlights the potential of virtual reality (VR) in telerehabilitation for individuals with cerebral palsy (CP), several limitations should be considered. (1) Specificity of the task: The study focused exclusively on a coincident timing task, which represents only one component of motor performance. The results cannot be generalized to other motor domains, such as functional reach or bilateral coordination. Further research is needed to determine whether similar effects occur in other types of motor skills. (2) Engagement and motivation: Psychological factors such as motivation and engagement were not directly measured. These factors could provide additional insights into the effectiveness of VR training and the observed improvements. (3) Control group assessment: The control group was assessed only once, which prevents direct longitudinal comparisons between groups. The control group was included to provide normative reference values for task performance, as the study was primarily designed to evaluate rehabilitation outcomes in individuals with CP. Consequently, multiple training sessions for participants without motor impairments would not have been clinically or ethically justified. (4) Sample size and statistical considerations: The study included a relatively small and specific sample, limiting generalizability. Due to the non-randomized design and the specific division of participants within CP diagnostic groupings, neither sample size calculation nor propensity score matching was feasible. Additionally, multiple statistical comparisons increase the potential risk of type I error. (5) Lack of blinding: Assessments were not blinded, which introduces the potential for observer or assessment bias. (6) Comparative analysis and generalizability across CP types: The study did not compare the VR intervention with other rehabilitation methods, limiting the ability to determine its specific advantages. Moreover, the results may not be generalizable to other forms of CP, such as hemiplegic or quadriplegic presentations. (7) Longitudinal evaluation across GMFCS levels: Differences in motor performance across GMFCS levels were only assessed on the first day of practice. Future longitudinal studies with larger samples are warranted to investigate whether CP severity influences the evolution of motor performance across repeated sessions.

### Future directions

To address these limitations, future research should focus on expanding sample sizes to enhance the generalizability of findings and incorporating a control group that undergoes VR training over multiple sessions to enable more comprehensive comparisons. Studies should integrate assessments of psychological factors such as motivation, enjoyment, and adherence, as well as the novelty effect of VR interventions, to better understand their role in motor learning outcomes and their contribution to the overall effectiveness of VR-based rehabilitation. Comparative analyses between VR-based telerehabilitation, traditional rehabilitation, and other technological interventions are needed to determine the most effective therapeutic strategies and the ecological validity of different approaches. Additionally, future research should investigate longitudinal performance changes across different GMFCS levels to provide insights into how individuals with varying motor abilities respond to VR-based training over time, helping to refine intervention protocols for personalized rehabilitation. Finally, exploring adaptive and multimodal VR systems designed to sustain motivation beyond short intervention periods (e.g., 10 days) and offering a variety of game-based tasks may optimize engagement, adherence, and performance improvements in telerehabilitation programs.

## Conclusions

This study demonstrates that home-based telerehabilitation using virtual reality is an effective approach for improving motor performance in individuals with cerebral palsy. Over the course of the 10-day intervention, participants exhibited significant improvements in both AE and VE, reflecting enhanced task performance. Additionally, an analysis of initial performance showed no significant differences between individuals with varying GMFCS levels, suggesting that VR-based rehabilitation allows for meaningful engagement across different functional abilities. While the control group outperformed the CP group, the consistent participation of individuals across all GMFCS levels highlights the accessibility of VR tasks and their potential to accommodate a wide range of motor impairments. These findings support the use of VR as a practical and adaptable rehabilitation tool for individuals with CP, reinforcing its potential for integration into home-based therapeutic programs.

## Supporting information

S1 Checklist CONSORT Checklist. (DOC)

S2 File Trial study protocol. (DOCX)

S3 Table Key Outcomes of the Virtual Reality Training Protocol: AE, VE, Hits, Effect Sizes, and Confidence Intervals. (XLSX)
