# Supplementary material for: Non-immersive virtual reality telerehabilitation for motor accuracy and precision in individuals with Cerebral Palsy: A non-randomized clinical trial
Source: PLoS One. 2026 Mar 13;21(3):e0343934. doi: 10.1371/journal.pone.0343934 (PMC12987460; doi:10.1371/journal.pone.0343934)
Supplement: S2 File — (DOCX) [file pone.0343934.s002.docx]

# ANALYSIS OF MOTOR PERFORMANCE IN A TASK PRACTICED IN AN ENVIRONMENT VIRTUAL THE DISTANCE (TELEREHABILITATION) IN PEOPLE WITH CEREBRAL PALSY

**SUMMARY**

**INTRODUCTION:** Cerebral palsy (CP) is defined as a permanent group of movement and posture disorders, varying in severity, causing limitations in activities of daily living and social participation. Individuals with CP require continuous monitoring and interventions to ensure that their development is not impaired and that they can adapt to daily activities. Interventions are commonly performed in rehabilitation centers, clinics specialized and demand time to displacement, resources financial and support family. Technological advancement and the growth of tools such as virtual reality and video conferencing applications became the telerehabilitation one modality what he can to be used node home. **OBJECTIVE:** The objective of this study is to analyze the feasibility and effectiveness of a home Telerehabilitation program using virtual reality in motor learning of children and young adults with CP. **METHODS:** Twenty volunteers diagnosed with CP aged between 5 and 40 years old will participate in the research, who after screening will be classified with the Classification System from the Function Motor Thick (GMFCS) and with the System of Classification from the Manual Ability (MACS). The motor task will be assessed with the MoveHero software, which assesses coincidental *timing* and it will be applied node 1st, 5th and 10th day of the protocol. You participants will perform 10 days of telerehabilitation with use from the reality virtual using the MoveHero. THE retention from the task motor, will be evaluated between the 15th and 30th day. For data analysis, performance measures related to the scope will be used. from the goal of task considering constant error and absolute error. The results will provide data for future interventions with remote technological resources in people with CP.

# INTRODUCTION

Cerebral Palsy (CP) involves a group of children who have movement and posture disorders, in addition to commonly presenting impairments in communication, speech ability, intellectual, disturbances musculoskeletal what harm the performance functional of the individual. Due to the presence of these dysfunctions, it is crucial that individuals with cerebral palsy have continued access to rehabilitation services (ROSENBAUN et al., 2006, COLVER, FAIRHURST, PHAROAH, 2014).

The ongoing need for care and interventions has a major financial impact, in terms of time and in managing the expectations of the individual and family. Furthermore, it is questionable whether commonly used interventions continue to add benefits when used repeatedly over the years or whether the gains are only punctual and short-term. There is a diversity of therapies proposed for individuals with cerebral palsy, however, not all of them demonstrate scientific evidence (NOVACK et al., 2013). According to Novack et al., among the interventions that receive strong indications for providing increased physical activity, functionality and self-care are home rehabilitation programs (NOVACK et al., 2013; NOVACK et al., 2020).

Despite of the advances what rehabilitation home it presents one tool what he can help a lot the evolution and implementation of therapies home and the use from the technology. To the new Digital tools can enable the use of information and communication technologies to provide rehabilitation therapy to people remotely, an intervention called home-based telerehabilitation (HRRT) (BRENNAN et al., 2010). HRRT includes a multicomponent approach, such as remote monitoring, e-learning, assessment, prevention, intervention, supervision, education, and consultation (GALEA, 2019; BATALIK et al., 2020). According to Szturm et. (2020), HRRT can provide continuous intervention treatment for individuals with CP, with the possibility of optimize you services of rehabilitation, reduce the time of therapist and allow for prolonged and regular practice at times convenient to users.

Of agreement with Llorens and al. (2015), you programs from the TRDC offer big flexibility for adapt schedules individual, they can release partially therapists of your schedules limited, reach areas remote where facilities clinics they can no be gifts, to save money expenses

related to transportation until the centers rehabilitation, and also adapt and monitor the distance therapy .

In the context of DCRT, the development of new interventions using technology such as virtual reality games becomes an interesting approach to find alternative treatments for various neurological difficulties, including cerebral palsy, and to continue rehabilitation or maintain its benefits. The use of VR can be considered an emerging approach to engage patients in the rehabilitation program and positively incorporate computer games in which a series of learning elements with interactive motor and cognitive challenges help individuals to participate in motor and repetitive activities, adaptive, meaningful and challenging task practice (LLORÉNS et al., 2015; SZTURM et al., 2020; SCHRODER et al. 2019). Rehabilitation games based on a virtual reality (VR) environment are accessible, motivating and whole-body movement practices and have potentially challenging options for a variety of rehabilitation clients (LEVAC et al., 2015).

You players they are motivated the produce movements bigger and the abandon the position sitting in front of the television or computer to be able to play these interactive games, which are activated through body movements (CROCETTA et al., 2015). In fact, the benefits of VR technology can be observed in children with cerebral palsy, improving gross motor function (ARNONI et al., 2019), positive results in gait performance, balance skills, strength muscular of the members lower (CHO and al., 2016), to improve the time reaction (POURAZAR et al., 2017), provide a greater amplitude and better control of the ankle dorsiflexion movement (BRYANTON et al., 2006), provide an opportunity for pleasant and motivating physical exercise and participation (YALON-CHAMOVITZ and WEISS, 2007).

Despite these benefits and the progression in the use of VR systems for rehabilitation and especially the combination of virtual reality in home therapy still need research to prove its effectiveness. Limited data, low methodological quality and high heterogeneity in the devices used (such as motion capture mode and different software) are factors that make it difficult to identify the benefits of telerehabilitation. According to (SCHOREDER et al. 2019), the way people with CP are trained at home and how learning in the clinic is transferred to the home environment make it difficult to draw uniform conclusions about the use from the telerehabilitation based in RV. One systematic review on you

telerehabilitation software systems performed at home with remote supervision (HOSSEINIRAVANDI et al 2020) presented three most common functional features for telerehabilitation (exercise plan management, reporting, and patient education). Therefore, monitoring, reporting, and optimizing physical behavior is important in the field of rehabilitation, and this approach requires valid and reproducible results to assess and monitor physical behavior and its changes in people with disabilities, even if they depend on a wheelchair (NOOIJEN et al., 2015). More than that, offering the opportunity for the rehabilitation team to determine and control the performance of the computer task of individuals with disabilities can help develop important strategies to maintain or improve their functionality (MALHEIROS et al., 2016).

Due to the COVID-19 pandemic in Brazil, on March 20, 2020, the Federal Council of Physiotherapy and Occupational Therapy (COFITTO), through Resolution No. 516, allowed the use of Teleconsultation and Telemonitoring modalities for physiotherapists and occupational therapists. This resolution arose from the need to continue interventions for patients who were undergoing treatment but had to be interrupted by the COVID-19 pandemic.

The interventions carried out were completely interrupted, or carried out more spaced out. Therapists (physiotherapists, occupational therapists, among other professionals) began to use resources such as guidance booklets and videos, activities and exercises to you country, with contacts carried out put platforms of meeting or video call. With the extension of the quarantine period, these practices end up becoming repetitive and uninspiring. Considering the modernity of the use of virtual reality and the need for telerehabilitation protocols (mainly due to the quarantine), the proposal of this research is to use a platform of games virtual to to check your effectiveness in the improve of performance in task motor in people with cerebral palsy. To this end, individuals with CP will undergo a telerehabilitation intervention protocol (10 sessions) with remote monitoring by the therapist to verify whether there will be an improvement in performance.

# OBJECTIVES

- 1. *OBJECTIVE GENERAL*

To evaluate the improvement in performance in a motor task practiced in a remote virtual environment (telerehabilitation) in people with cerebral palsy.

- 1. *OBJECTIVE SPECIFIC*
     - Characterize the functional, sociodemographic and technological profile of people with paralysis cerebral.
     - Characterize the performance in the task of *timing* coincidental and people with paralysis cerebral.
     - Compare the performance in the task of *timing* coincidental between you different levels of cerebral palsy.
     - Compare the performance in the task of *timing* coincidental before and after the realization of the telerehabilitation protocol.
     - To check if exists retention of performance from the task carried out node environment virtual from a distance.

# METHODS

- 1. *DESIGN DO STUDY*

This is a research project whose clinical trial is longitudinal, uncontrolled, prospective, accomplished with people with paralysis cerebral between 5 the 40 years, what will participate of a home telerehabilitation program in a virtual environment.

The sample will be characterized using motor and manual classification systems for people with cerebral palsy and sociodemographic and technological profile questionnaires.

The proposal is to verify whether there is an improvement in performance in a motor task when practiced in environment virtual the distance (telerehabilitation). To such analysis it will be used one virtual reality game carried out at home, with remote monitoring by the researcher.

To so much, it will be used a task of coincidental *timing* evaluated with the game MoveHero, which it will be also the tool of practice virtual put 10 days. You results of *timing* coincidental of the 1st, 5th and 10th days of game will be compared to analysis of performance engine in the task. After After 30 days, people will be evaluated again to analyze the retention of results.

- 1. *PARTICIPANTS*

The sample will be formed by individuals who, through a detailed analysis, meet the eligibility criteria for this study and agree to participate in the study by signing the Free and Informed Consent Form (FICF) (ANNEX 1) and the Minor's Assent Form (ANNEX 2), both previously approved by the Research Ethics Committee.

Then, the research participants will be analyzed through a Sociodemographic and Technological Profile Questionnaire, followed by the classification of gross motor function and manual ability carried out by interview with parents and video analysis.

The sample size was calculated using statistical software (GPower 3.1.5) for the main outcome measure (i.e., motor score). This calculation was based on data from a study with a group of individuals with CP (Duarte et al., 2014) and a test power of 0.80 was used; the alpha he was of 0.05; and the size of effect he was 0.65 (d of Cohen). THE I estimated from the sample indicated that 15 participants (performing 10 interventions each one) would be necessary. With one adjustment To allow for a withdrawal rate (20%), we will recruit 20 participants.

Twenty volunteers aged between 5 and 40 years old, diagnosed with cerebral palsy and their guardians, recruited from among those who attend the FHO- Araras (SP) Physiotherapy School Clinic and the specialized Neurorehabilitation clinic, Intensive Care Clinic, will participate in the study.

- 1. *CRITERIA OF INCLUSION AND EXCLUSION*

The following inclusion criteria will be considered: (1) they agree to participate in the research, signing the Assent Form, and their legal guardians, signing a Free and Informed Consent Form; (2) have a clinical diagnosis of CP performed by a neuropediatrician (informed by the parents); (3) with GMFCS and MACS between levels I and V; (4) age ranging from 5 to 40 years.

Participants will be excluded if (1) they do not understand the game tasks - understanding the task it will be evaluated after 2 minutes of practice (the individual it will be deleted if no to understand the task during you first two minutes of practice); (2) present difficulties motors what prevent the realization of the tasks virtual (the individual it will be deleted if no to present capacity motor to perform the task for 2 minutes); (3) surgery or use of an upper limb spasticity inhibitor during the last six months; (4) not having technology devices to perform telerehabilitation (computer or tablet) and (5) inability to complete the task due to some technological failure (such as Internet or computer failure).

# PROCEDURES

THE design of this study he can be summarized with the figure 1.

THE study it will be divided in 4 steps. THE first stage contempla the characterization from the sample with data clinical and functional, profile sociodemographic and technological. THE second stage includes the evaluation of the motor performance performed by the task at the coincidental *timing* performed by the MoveHero software for 3 minutes, with 3 evaluations being carried out. The third stage consists of carrying out of 10 days of training of practice motor with reality virtual the distance, and in the fourth stage the reassessment of motor performance in the coincident *timing task* after 30 days of completing the remote practice. Between 15 and 30 days, the retention assessment will be carried out.

**1st STAGE - Sample characterization**

**GMFCS, MACS, technological profile**

**2nd STAGE - Evaluation of coincident timing**

**Time: 3 minutes, Number: 3 times**

**3rd STAGE - 10 days of virtual reality training**

**Time for each practice: 10 minutes per day**

**4th STAGE - Reassessment of coincident timing**

**Time: 3 minutes, Number: 3 times**

**5th STAGE - Retention**

**Reassessment of coincident timing**

Figure 1 - Design of study with the description of the steps from the search.

# PROTOCOL EVALUATION​

This study aims to analyze the motor performance of people with cerebral palsy when subjected to a virtual reality training protocol carried out remotely. Each participant will be evaluated using the instruments described below.

- - 1. *Questionnaire sociodemographic and of general conditions of health*

To collect data, a questionnaire containing sociodemographic indicators such as age at years, sex, conditions associated with paralysis brain, therapies and activities carried out and the time put week spent in these activities, income familiar in minimum wage, parents' years of education, number of people in the same residence. Regarding the technological profile, the following will be collected: dominant hand, devices used, screen time, familiarity with video games and use of virtual reality in therapy.

All you instruments will have version of form electronic to facilitate the analysis of data.

- - 1. *Profile engine and manual skill*

To characterize the sample of study, and provide the analysis of the possible influences of the conditions related to functionality in the motor performance results, participants will be classified according to gross motor function with the Gross Motor Function Classification System (GMFCS) and as the function manual with the System of Classification from the Ability Manual (MACS), both were developed to categorize, respectively, the mobility and manual function of children with CP (PALISANO et al, 1997; ELIASSON et al., 2006) and are characterized as an ordinal scale of five levels that portray, in decreasing order, the level of independence and functionality of children with CP.

*System of Classification of Motor Function Gross (GMFCS)*

THE GMFCS and system of classification standard reliable and valid from the function motor thick for cerebral palsy that analyzes an individual in five levels, according to their ability to move, limitations functional, the need of to use devices auxiliaries to to walk or the need for a wheelchair (GONZÁLEZ-ALONSO; PALISANO et al. 1997). The GMFCS is based on voluntarily initiated movement, emphasizing sitting and walking. The distinctions between levels of motor function, from I to V, are based on functional limitations and the need for assistive technology. Despite the age range established up to 18 years, there are already studies in the literature that use the GMFCS for adults with CP (SANDSTROM, ALINDER AND OBERG, 2004).

THE classification it will be carried out through from the analysis of video of the participants and confirmed with therapists/parents through interviews.

*System of Classification of Skill Manual (MACS)*

MACS describes how children with CP use their hands to manipulate objects in everyday activities and describes five levels based on the child's ability to initiate manipulation on their own. of objects and the need of assistance or adaptation to carry out activities manuals

in the life daily. THE MACS evaluates the ability global from the child in the manipulation of the objects node everyday life, does not consider differences in function between the hands; instead it addresses the way children manipulate age-appropriate objects (ELIASSON et al., 2006). In the MACS, regardless of age, children who are able to manipulate objects easily are classified as in level I and those what manipulate objects with minor quality belong to the II. Already the children of level III, manipulate objects with difficulty needing of help or adaptation of the activity and, node IV, they are those what execute activities manuals with success limited, requiring continuous supervision. Finally, level V includes children with severely impaired manual skills, requiring full assistance (MORRIS et al., 2006)

- - 1. *Assessment of Timing Coincident*

timing is the perceptual-motor ability to execute a motor response in synchrony with the arrival of an external object at a given point (HART and REEVE, *1997* ). CT depends on selection, planning and execution processes to anticipate both the arrival of the stimulus and the effector response so that they occur simultaneously (TRESILAN, 1995). Traditionally, to the measures of performance used in studies with tasks of *timing* coincidental they are: the error absolute, what express the magnitude of error; the error constant, which expresses the direction of the error (late or early responses) and the variable error, which expresses the consistency of performance (SCHIMIDT and LEE, 1999). During practice, when absolute errors, constant and variable decrease and one clue what the system perceptual-motor it found an appropriate solution to solve your temporal organization problem, and tends to repeat that solution.

timing will be assessed with MoveHero software, as well *as* remote virtual reality training.

# REMOTE VIRTUAL REALITY ASSESSMENT AND TRAINING WITH THE MOVEHERO GAME

The MoveHero Software was developed at the School of Arts, Sciences and Humanities at the University of São Paulo and is used both to assess coincidental *timing* and as an intervention instrument in virtual reality.

THE objective of MoveHero, and intercept to the balls what fall in four positions different, at the exact moment that reach a target (goal). The game features the silhouette of your image (avatar) and four rings (targets), two on each side of the computer screen, and the participant must intercept the balls what fall in these four positions, node rhythm of one music, moving to the hands in the in front of a computer camera ( *webcam* ) (MARTINS et al., 2019;).

Attempts to touch the ball before it hits one of the rings are scored as *early attempts* , to the balls what pass by the rings without to be touched for the participant they are registered as *lost* , and to the correctly touched in any position within of the rings as *hits.* The time in milliseconds of the ball touch is calculated as the time interval between the target time (equal the 0, when exactly within from the ring) less the time of touch. That time he can be negative, when the ball is touched before it is exactly in the center position (early), or positive, when the ball is touched after it has passed the center point (late). The coincident *timing* is stored with the ball position indicators (A, B, C and D), allowing analyses to be carried out based on the laterality of the balls' reach.

THE participant he must synchronize the movement of the hands with the moment in what to the balls that fall if they meet in the circles, therefore, it requires bilateral movements with the need for precision to the right and left. As *feedback* for success or failure, the game offers visual and sound effects.

When the participant execute correctly the reach, the color of the balls change to green,

indicating hit, or to red, indicating error, when the ball get out of circle without being touched or when it is reached early in addition to a beep indicating an error.

The duration of the game is chosen by the researcher, as is the music that accompanies the game. and the sequence of the balls. THE game MoveHero he has 10 phases, and in each phase the degree of difficulty occurs as the number of balls increases. The score is given by the number of balls correctly reached by the participant (hits) and shown to him, multiplying the result of number of hits put 10. Like this, each ball correctly achieved receives 10 points. The game also stores the number of balls that fall without being touched (lost) and the number of balls touched before reaching the respective ring (anticipated) (GUARNIERI et al., 2018). At the end of the game, a screen opens with the total score achieved in the match.

*timing* data used as a parameter of motor performance, the absolute error, the constant error and the variable error, which expresses the consistency of performance, will be collected on the 1st, 5th and 10th day of remote virtual reality intervention. And retention will be evaluated after 30 days after the end of the protocol.

In each evaluation will be carried out 3 measures with each one having 3 minutes of duration.

# REALITY VIRTUAL TO DISTANCE (TELEREHABILITATION)

Once the sample has been characterized and evaluated, the application of the reality protocol will begin. virtual the distance during 10 days. Each intervention he has approximately 10 minutes application and another 5 to 10 minutes of preparing the environment to carry out the practice.

Before starting to apply virtual reality remotely, it will be necessary to carry out training of familiar responsible of driving from the practice node home. This training precedes practice and consists of 3 phases, described below.

- 1. *GUIDELINES AND TRAINING TO THE REALITY VIRTUAL THE DISTANCE*

Before we begin applying the protocol, it will be necessary to provide some guidance and training to the person responsible for conducting the practice.

- - 1. *Environment choice and security*

A location in the home for virtual reality practice should be chosen with good lighting and no details (paintings, windows, shelves) to allow for adequate capture of images by the webcam. The chosen location should have a minimum space of 1.5 meters between the table where the laptop should be placed and the participant, the location should not have carpets and be away from corners.

One time that the person in charge selects the necessary space and equipment, the The researcher can confirm whether it is suitable by requesting a video scan of the chosen and prepared environment.

- - 1. *Familiarization with the MoveHero*

The platform called MoveHero will be used, available for free use at https://movehero.com.br/. THE participant will need access the Internet and, one time online, should create your own account, enter your name, email and create a password. In case of difficulty accessing the network of Internet, or equipment with minor capacity of memory, it will be used the game installed in the notebook. In this case, the family will receive the guidance put quite of one video presenting the step by step guide to installing the game ( <https://youtu.be/OgEUPeclf1M>). Considering that the platform has different levels of difficulty, after the participant is connected, the researcher will perform the targeting of participant to the protocol especially developed to the cerebral palsy and will training of the person responsible for monitoring telerehabilitation to use of the platform in terms of game settings, choice of phases, camera activation and positioning of the notebook in relation to the participant.

The following instructions using text message, or email or video call will be carried out:

1. put the computer in a table and do login to platform;
2. check it out the settings of game, what he must be in level average, error to the anticipate activated, and camera activated, mandatory.
3. position the telephone cell phone (to to supply video call) in the side of computer to receive instructions;
4. the researcher will explain the task verbally to all participants and ask the family member to play (demonstrations of how to perform the coincidence time were given by the family member for two minutes);
5. After the demonstration, the family member must position the cell phone in a way that allows the researcher to see the participant's performance throughout the protocol;
   - 1. *Positioning and training*

THE position to realization of game it will be chosen of agreement with the level from the GMFCS of the participant.

- - - - Level I and II: Standing
      - Level III: In foot with support or sitting in chair (according to assessment clinic)
      - Level IV and IV: Sitting on the chair of wheels

The participant must be positioned at a distance of approximately 1.5 meters from the notebook monitor and the camera must be adjusted so that the game balls are reached by the moving upper limbs, and that the targets do not touch the shoulders or head. To determination from the distance ideal to each participant, it will be requested what perform a shoulder abduction to analyze whether the hands reach the target rings. As there is variation related to the range of motion, muscle tone and motor coordination of the upper limbs of people with CP, the ideal adjustment will be made on an individual basis.

By video call, the researcher will instruct the participant to stand still and wait for the first sphere to appear on the screen, while the person in charge will start the game. Once the first sphere appears on the screen, the participant will be instructed on when to move their hand. in direction to the target. It will be used the instruction "Attention what to the balls they go to start the to fall", and will assist the participant throughout the first game with verbal instructions.

# PROTOCOL

Once the sample has been characterized and the *timing* coincidence assessed, remote training with the MoveHero Game will begin, consisting of 10 days of training. On the 5th ^and^ 10th ^days^ , the *timing* coincidence will be reassessed, and after 15 to 30 days, the learning retention will be assessed. All assessments will be conducted using the MoveHero Game itself (Figure 2).

Each training day is divided into 3 three-minute matches, totaling approximately 10 minutes of activity.


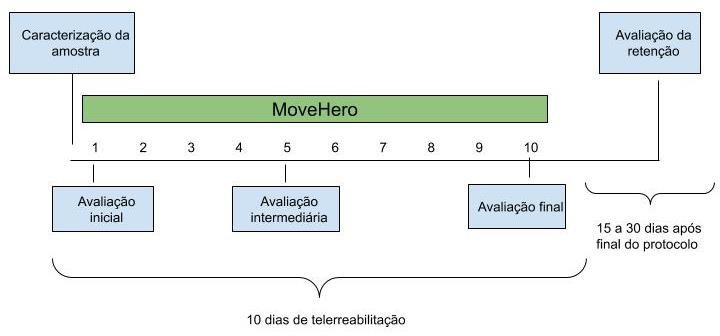


Figure 2 – Scheme of the training protocol with reality virtual at a distance.

# METHODOLOGY OF ANALYSIS OF DATA

To analyze the data, descriptive statistics will be performed to characterize the sample and the results will be presented with mean and standard deviation values.

For inferential analysis of the coincident *timing task* practiced, the following variables will be analyzed: dependents to the measures of error in milliseconds (errors constants what evaluates the directional tendency of the movement, absolute that demonstrates the accuracy of movement and Variable that identifies the precision of the movement). If the data meets the assumptions for using analysis parametric, it will be carried out analysis of variance (ANOVA), to identification of differences. Differences, if any, will be detected by the Tukey-HSD post-hoc test. If the normality assumptions are not met, non-parametric analyses will be used to identify and locate differences: Friedman – post-hoc Wilcoxon test (intra-group) and Kruskal-Wallis and Mann-Whitney U as post-hoc test (between groups). A level of significance of 0.05 (5%) and all you intervals built to the far away of work will be with 95% statistical confidence. The statistical program will be SPSS *(Statistical Package for Social Sciences* ), version 20.0.

# FEASIBILITY OF THE PROPOSAL

The research project will be developed in partnership with Prof. Dr. Carlos Bandeira de Mello Monteiro, professor of the Department of Postgraduate Studies in Rehabilitation Sciences at the School of Medicine and the School of Arts, Sciences and Humanities (EACH), and the Physical Education and Health Program at the University of São Paulo. This professor will facilitate the use of equipment and the development and maintenance of virtual games. The researcher responsible for preparing and conducting the study is qualified to perform the therapeutic intervention.

# RISKS AND BENEFITS

*RISKS*

You risks they are considered minimum, as discomfort what may to feel as to stay tired, to stay nauseous (the), no get to execute the movement necessary to what the game it is carried out. If he (the) to feel any type of discomfort, may to warn the researcher and to stop immediately to carry out the games, without any harm. During the games, those responsible will be instructed to remain next to the participant at all times. Participants who are unbalanced will carry out the training sitting down to avoid falling, and participants who do not have trunk control will carry out the activities sitting down in their own wheelchair, which is safe and has specific adaptations for each case.

*BENEFITS*

The benefits of participating in this telerehabilitation protocol are improved upper limb movement, trunk balance and weight-bearing on the lower limbs. (to you what may carry out in foot), increase of level of activity physical and knowledge of a virtual reality tool suited to the profile of people with disabilities. After the protocol is completed, the tool will be available for recreational use by the participant and family, and clinical therapists will also be able to use this tool to enhance therapies households. As form indirect, the search will help the validation scientific of activities

proposals in virtual reality games that will help many people with disabilities or difficulties in movement.

# RESULTS EXPECTED AND CHALLENGES

It is expected that telerehabilitation using virtual reality will bring improvements in motor performance (motor learning) of people with cerebral palsy. If this result is achieved, it will be possible to use the knowledge acquired in this research for organization and planning of therapies home what come the contribute with the rehabilitation of these individuals, in addition to enabling home therapy monitoring, prolonged intervention practice, and reduced travel time and costs to rehabilitation sites.

As a challenge for the development of the research, we can list the need for a digital tool for remote communication, the development of new learning for the use of virtual reality with the game that will be offered, in addition to the availability of the family (caregiver) to monitor the intervention together with the researcher, which takes time and organization of the family routine.

These difficulties will be expired with the training of family member/participant to the use of the tools before of start of protocol of search, carried out of form remote, and organization appropriate periods for carrying out the research according to the routine of the participant and family.

# ASPECTS ETHICAL AND LEGAL OF SEARCH

This search it will be submitted to the Committee of Ethics in Search from the Faculty of Medicine of the University of They are Paul. All you participants of search will receive explanations oral and written information about the study and, upon agreeing, they will sign the TCLE which will be prepared in two copies, one being retained for the subject from the search or put your representative legal and one archived for the researcher. The TCLE presents information about all procedures to be carried out, as well as the rights guaranteed to participants. To this end, Resolution No. 466, of December 12, will be respected. of 2012 (BRAZIL, 2012) of Advice National of Health/MS what regulates research involving human subjects and the Declaration of Helsinki (1964). Data will be stored electronically in bases of data with access restricted and safe. All the data will be coded with removal of any information that could identify individuals.

# REFERENCES

ARNONI, JLB, et al. Effects of virtual reality on body oscillation and motor performance of children with cerebral palsy: A preliminary randomized controlled clinical trial. *Complementary therapies in clinical practice* , v. 35, p. 189-194, 2019.

BATALIK, L. et al. Benefits and effectiveness of using a wrist heart rate monitor as a telerehabilitation device in cardiac patients: THE randomized controlled trial. *Medicine* , v. 99, n. 11,

p. e19556, 2020.

BRENNAN, D., TINDALL, L., THEODOROS, D. et al. A blueprint for telerehabilitation guidelines. *International journal of telerehabilitation* , v. *2, no.* 2, p. 31–34, 2010. <https://doi.org/10.5195/ijt.2010.6063>

BRYANTON, C., et al. Feasibility, Motivation, and Selective Motor Control: Virtual Reality Compared to Conventional Home Exercise in Children with Cerebral Palsy. *Cyber Psychology & Behavior,* v. 9, no. 2, p. 123–128, 2006. doi:10.1089/cpb.2006.9.123

CHO, W. and al. Treadmill Training with Virtual Reality Improves Gait, Balance, and Muscle Strength in Children with Cerebral Palsy. *Tohoku J Exp Med* 2016;238(3):213–218. doi:10.1620/tjem.238.213.

COLVER, THE., FAIRHURST, W., PHAROAH, P. THE. D. Cerebral palsy. *The Lancet,* v.383, n. 9924, p.1240–1249, 2014. doi:10.1016/s0140-6736(13)61835-8

CROCETTA, TB, et al. Virtual and augmented reality technologies in Human Performance: a review. *Physiotherapy in Movement* , v.28, p.823-835, 2015.

ELIASSON AC; et al. The Manual Ability Classification System (MACS) for children with cerebral palsy palsy: scale development and evidence of validity and reliability. *Developmental Medicine and Child Neurology,* vol. 48, p.549-554, 2006.

GALEA, MD Telemedicine in Rehabilitation. *Phys Med Rehabil Clin N Am* . v.30, n. 2, p.473– 483, 2019. doi:10.1016/j.pmr.2018.12.002

GONZÁLEZ-ALONSO, MY, MAT[IA CUBILLO, AC Characteristics of users of the Cerebral Paralysis Association. Family Medicine. SEMERGEN, 2018. doi:10.1016/j.semerg.2018.07.003

GUARNIERI, R. et al. Assessment of movement anticipation ability in children and adolescents with intellectual disabilities. DI Journal, n.13-17, p.35-40, 2018.

HART, M.A., REEVE, T.G.A. preliminary comparison of stimulus presentation

HOSSEINIRAVANDI, M. et al. Home-based telerehabilitation software systems for remote supervision: THE systematic review. *International Journal of Technology Assessment in Health Care,* vol. *36, no.* 2, 113-125, 2020. doi:10.1017/S0266462320000021

LEVAC, D., et al. "Kinect-ing" With Clinicians: A Knowledge Translation Resource to Support Decision Making About Video Game Use in Rehabilitation. *Physical Therapy* , v.95, p. 426-440, 2015.

LIORÉNS, R., et al. Effectiveness, Usability, and Cost-Benefit of a Virtual Reality–Based Telerehabilitation Program for Balance Recovery After Stroke: A Randomized Controlled Trial. Archives of Physical Medicine and Rehabilitation, v.96, n. 3, p.418–425, 2015. doi:10.1016/j.apmr.2014.10.019.

MALHEIROS, S. A. Computer task performance by subjects with Duchenne muscular dystrophy.

*Neuropsychiatr Dis Treat* , 12, 41-48, 2016.

MANCINI, MC *Pediatric Disability Assessment Inventory (PEDI):* adapted Brazilian version manual. Belo Horizonte: UFMG, 2005.

MARTINS, FPA, et al. Analysis of motor performance in individuals with cerebral palsy using a non-immersive virtual reality task - the pilot study. *Neuropsychiatr Dis Treat* . v.15, p.417–428, 2019.

MORRIS, W.; KURINCZUK, JJ; FITZPATRICK, R.; ROSENBAUM P. L. Reliability of the

manual ability classification system for children with cerebral palsy. *Dev Med Child Neurol.* v.48,

n. 12, p.950-3, 2006.

NOOIJEN, W. F. J., and al. Validation of an activity monitor for children who are partly or completely wheelchair-dependent. *Journal of Neuroengineering and Rehabilitation* , v.12, n.11, 2015. doi.org/10.1186/s12984-015-0004-x

NOVACK et al. (2020). State of the Evidence Traffic Lights 2019: Systematic Review of Interventions for Preventing and Treating Children with Cerebral Palsy. *Current Neurology and Neuroscience Reports.* v.20, n.3, p.2-21, 2020. doi.org/10.1007/s11910-020-1022-z.

NEWK, I. and al. THE systematic review of interventions for children with cerebral palsy: state of the evidence. *Dev Med Child Neurol* . v.55, n. 10, p.885‐910, 2013 doi:10.1111/dmcn.12246

PALISANO, R, et al. Development and reliability of a system to classify gross motor function in children with cerebral palsy. *Dev Med Child Neurol* . v.39, n. 4, p. 214–223, 1997.

PALISANO, R. et al. Development and reliability of a system to classify gross motor function in children with cerebral palsy. *Dev Med Child Neurol* . v.39, n.4, p.214-23,1997.

PARMANTO, B. and al. Development of the telehealth usability Questionnaire (TUQ). *International Journal of Telerehabilitation.* v.8, n.1, p.3-10, 2016.

TO POUR, M. and al. Use of virtual reality intervention to improve reaction team in children with cerebral palsy: A randomized controlled trial. *Developmental Neurorehabilitation* , v.1, n.6, 2017. doi:10.1080/17518423.2017.1368730

ROSENBAUM, P. and al. THE report: the definition and classification of cerebral palsy April *Dev Med Child Neurol Suppl* 2007; v.109, p.8–14, 2006.

SANDSTROM, K; ALINDER, J.; OBERG B. Descriptions of functioning and health and relations to a gross motor classification in adults with cerebral palsy. *Disabil Rehabil* . v. 26, no. 17, p. 1023- 31, 2004.

SCHMIDT, FROG, READ, TD *Engine control and learning* : the behavioral emphasis. 3 ed. Champaign: Human Kinetics, 1999.

SCHRÖDER, Jonas et al. Combining the benefits of tele-rehabilitation and virtual reality-based balance training: the systematic review on feasibility and effectiveness. *Disability and Rehabilitation: Assistive Technology,* v. 14, no. 1, p. 2-11, 2019.

SZTURM, T., and al. Evaluation of the game based Tele rehabilitation platform for in-home therapy of hand‐arm function post stroke: Feasibility Study *. PM&R.* (2020). doi:10.1002/pmrj.12354.

TRESILIAN, JR Perceptual and cognitive process in time-to-contact estimation. *Percept Psychophys.* v.57, n.2, p.231-245, 1995.

YALON-CHAMOVITZ, S; WEISS, PL (Tamar). Virtual reality as a leisure activity for young adults with physical and intellectual disabilities. *Research in Developmental Disabilities* , v.29, n.3,

p. 273–287, 2008. doi: [10.1016/j.ridd.2007.05.004](https://doi.org/10.1016/j.ridd.2007.05.004)
